# Pre-Exposure Prophylaxis (PrEP) Uptake among Black Men Who Have Sex with Men (BMSM) in the Southern U.S.

**DOI:** 10.3390/ijerph18189715

**Published:** 2021-09-15

**Authors:** Oluwafemi Adeagbo, Sayward Harrison, Shan Qiao, Xiaoming Li

**Affiliations:** 1 South Carolina SmartState Center for Health Care Quality, Arnold School of Public Health, University of South Carolina, Columbia, SC 29208, USA; HARRI764@mailbox.sc.edu (S.H.); shanqiao@mailbox.sc.edu (S.Q.); xiaoming@mailbox.sc.edu (X.L.); 2 Department of Health Promotion, Education & Behavior, Arnold School of Public Health, University of South Carolina, Columbia, SC 29208, USA; 3 Faculty of Humanities, University of Johannesburg, Johannesburg 2006, South Africa; 4 Department of Psychology, College of Arts and Sciences, University of South Carolina, Columbia, SC 29208, USA

**Keywords:** BMSM, HIV, PrEP, United States, Southern states, COVID-19

## Abstract

Black men who have sex with men (BMSM) living in the United States (U.S.) South are disproportionately affected by HIV and experience significant disparities in HIV incidence, access to HIV care, and prevention across ages and socio-economic statuses. The aim of this commentary is to critically review current literature on the state of PrEP use among BMSM in the U.S. South, including identifying barriers and facilitators to PrEP use in order to inform intervention development. Extant literature shows that despite the documented benefits of PrEP as an effective HIV-prevention method, its uptake among BMSM is limited across the U.S. South. Common barriers to PrEP uptake included stigma, homophobia, mistrust of healthcare systems, negative attitudes from healthcare providers, access and transportation issues, poverty, and misinformation about PrEP. These barriers are likely to have been further exacerbated by the COVID-19 pandemic. Limited access to PrEP and other HIV-prevention programs, such as HIV testing, post-exposure prophylaxis (PEP), and condoms for BMSM are likely increase HIV incidence in this community. Moreover, the rapid expansion of telehealth services during the COVID-19 period may offer increased opportunity to scale-up PrEP through telehealth interventions, especially if in-person services remain limited due to pandemic precautions. Given the intersectional barriers that limit the access and uptake of PrEP among BMSM, we suggest that tailored programs or interventions that seek to address PrEP disparities among Southern BMSM should adopt intersectional and interdisciplinary approaches to better understand the complex challenges of scaling up PrEP. More studies are needed to investigate the impact of COVID-19 on HIV-prevention services among BMSM and to understand how to co-develop—with the BMSM community and healthcare providers—culturally acceptable interventions to reduce the identified challenges using intersectional and interdisciplinary approaches.

## 1. Introduction

The United States (U.S.) federal government launched the “Ending the HIV Epidemic: A Plan for America” (EHE) initiative in 2019 with the goal of ending the HIV epidemic by 2030 [1]. Six of the seven states identified as key statewide targets in the EHE initiative due to a disproportionate occurrence of HIV in rural areas are in the South [2,3]. The South is home to 38% of the U.S. population but accounted for more than half of all new HIV diagnoses in 2018 [4]. Additionally, the rate of AIDS diagnosis (10.3 per 100,000) was higher in the “Deep South” than in the U.S. general population (6.7 per 100,000) [3]. The EHE initiative prioritizes wider pre-exposure prophylaxis (PrEP) coverage, especially among populations at higher risk of HIV, including Black men who have sex with men (BMSM) [1,3,5]. Thus, the aims of this article are to review current empirical studies and epidemiologic data on the state of PrEP use among BMSM in the Southern states and to provide recommendations for future research and programmatic directions. We adopt the Centers for Disease Control and Prevention (CDC) designation of the Southern U.S., which comprises the District of Columbia and 16 states: Alabama, Arkansas, Delaware, Florida, Georgia, Kentucky, Louisiana, Maryland, Mississippi, North Carolina, Oklahoma, South Carolina, Tennessee, Texas, Virginia, and West Virginia.

### Disproportionate Impact of HIV on BMSM

Black MSM are disproportionately affected by HIV, including experiencing significant disparities in HIV incidence as well as in access to HIV care and prevention across ages and socio-economic statuses [5,6,7,8]. For example, a meta-analysis that compared disparities and risks of HIV infection among MSM in the U.S., United Kingdom, and Canada found greater poverty and lower rates of health insurance among BMSM in the U.S. than in the UK and Canada [9]. Black MSM living in the U.S. South—especially those in rural areas—may experience particular access barriers and healthcare systems issues—including systemic racism—that negatively affect their health outcomes [3]. Of the estimated 36,801 new HIV diagnoses in the U.S. in 2019, 69% were among men who have sex with men (MSM), and 37% were among BMSM [10]. According to the CDC, one in two BMSM will be diagnosed with HIV in their lifetime, if current trends persist [11]. Forty percent (40%) of young BMSM will be infected with HIV by age 30, according to the HIV-prevention trials network (HPTN) 061 [12]. Generally, MSM are 83% times likely to be infected with HIV than heterosexual men due to individual, interpersonal, and structural risk factors [7,11]. Estimates suggest that BMSM in the South are five times more likely to acquire HIV than their White counterparts [6,13]. Black MSM living in Southern states are less likely to be tested for HIV; if HIV-positive, they are less likely to initiate antiretroviral treatment (ART), be retained in care, and achieve an undetectable viral load when compared to White MSM [3,5]. The use of antiretrovirals for PrEP is a safe and highly effective method that has been demonstrated through multiple clinical trials to prevent HIV [3,6,14,15]. The U.S. Food and Drug Administration (FDA) has approved the use of Truvada (tenofovir disoproxil fumarate/emtricitabine) and Descovy (emtricitabine 200 mg and tenofovir alafenamide 25 mg) for PrEP to prevent HIV, with both medications clinically indicated for use among MSM [6,16]. Despite the benefits of PrEP, evidence indicates there is poor uptake of PrEP across the U.S., especially among BMSM [3,5,7,14,15].

## 2. BMSM PrEP Uptake in the South

Prior studies in the U.S. have investigated trends in PrEP use across different regions [3,7,17,18,19]. A recent analysis of 3108 counties across the U.S. showed that PrEP use was slightly higher in 2018 when compared to 2012 [7]. However, lowest prevalence of PrEP uptake was observed primarily in states in the South: Georgia, Kentucky, North Carolina, South Carolina, Virginia, and West Virginia [7]. Noticeable gaps exist between the willingness to use PrEP and its uptake across the U.S. [3,5,7,8]. Between 2014 and 2015, 68.8% of 333 BMSM surveyed in the American Men’s Internet Survey were willing to use PrEP, but only 7.5% used PrEP in the previous year [20]. Similarly in 2017, a national behavioral survey conducted in 23 U.S. cities revealed that 78.3% of BMSM were aware of PrEP, but only 18.8% had used it [21]. A recent national survey conducted with young BMSM (*n* = 147) revealed that 52% of participants reported that they were likely to be infected with HIV, 39% had heard about PrEP, and 62% disclosed their sexual orientation to healthcare providers and were willing to take PrEP; however, only 8% reported having taken PrEP [22]. In a study conducted with 778 HIV-negative MSM in Atlanta, Detroit, and New York City, 31.2% reported currently taking PrEP, 7.6% previously used it, and 61.2% had never used PrEP due to health concerns (safety), cost, availability, and individual needs [23]. To close the gaps between PrEP knowledge, willingness, and actual usage among BMSM, efforts must be directed towards identifying specific barriers to PrEP uptake and address these barriers through tailored interventions and PrEP campaigns for BMSM given that only a few of the extant strategies to improve PrEP awareness and uptake focus on BMSM [5,6,7].

Surveillance data from 2015 showed that an estimated 1,144,550 million adults had indications for PrEP use [8]. The majority of these “eligible candidates” for PrEP use were MSM (71.1%) when considering likely sources of HIV risk, with Black individuals (43.7%) having the highest indications for PrEP use across races [8].The indication (i.e., PrEP eligibility) for adult MSM was based on individual’s negative HIV serostatus diagnosis, being sexually active with multiple male partners, and having exposure to risks (e.g., unprotected anal sex) that could increase their chances of acquiring HIV. For the current review, we extracted the Southern states’ data (see Table 1 and Table 2) from nationally representative CDC data to show the number of eligible adults and BMSM who had indications for PrEP in 2015. Table 1 shows the total number of all adults in the general population and the estimated number of HIV-negative MSM with PrEP indications (by transmission risk group) across the 16 Southern states and the District of Columbia. Table 2 indicates the total number of Black/African American adults as well as total number of BMSM with PrEP indications (by race/ethnicity) in the South. Indications for PrEP use was high among BMSM (60%) compared to all Black/African American adults who were estimated to be good candidates for PrEP in the South. However, the analysis of 2017 nationally representative data showed poor uptake of PrEP in the Southern U.S. [3]. The high number of PrEP indications among Southern adults—particularly BMSM—did not translate to PrEP uptake across the Southern states, as shown in Table 3. For example, of the 9040 adults in South Carolina who had indications for PrEP, only 590 (6.5%) were prescribed PrEP in 2017 [3]. Thus, while the South has the highest proportion of people living with HIV and higher number of PrEP indications, there is low PrEP uptake [7,24]. This shows that many of those who could potentially benefit from PrEP are not in care and suggests that current national goals to end the HIV epidemic will be difficult to achieve without concerted efforts to enhance PrEP use among Southern individuals at risk for HIV.

### Barriers to PrEP Uptake among BMSM in the U.S. South

Stigma is a major barrier to PrEP initiation among BMSM who may anticipate and/or encounter negative experiences with healthcare providers, such as being labelled as “promiscuous” when attempting to obtain PrEP [6,25,26]. Recent studies reported that BMSM are less likely to access PrEP and less likely to disclose their sexual identity and sexual behaviors to healthcare professionals due to stigma [16,22,27,28]. HIV-related stigma is high in the South, and PrEP could be associated with HIV risk or an HIV-positive status, which furthers create barriers for uptake [8,26,29]. Other barriers to effective uptake of PrEP among BMSM include mistrust in healthcare systems and socio-structural barriers, such as poverty, racism, and homophobia [5,8]. For example, BMSM in the South are disproportionately affected by poverty, and the refusal of many Southern state legislatures to expand Medicaid benefits to low-income residents has exacerbated existing health and healthcare disparities [14,30]. Additionally, one in eight MSM eligible for PrEP in the South lived between 30–60 min’ drive from the providers, and access issues due to transportation are common [3]. Furthermore, there are inconsistencies in how providers assess HIV risk factors and PrEP candidacy among BMSM [5,31]. For example, a 2017 study conducted in six Southeast states among healthcare providers (*n* = 820) revealed that 52.3% had no knowledge of PrEP, and only 18.1% had prescribed PrEP [32]. A recent study from South Carolina revealed similar findings, where healthcare providers missed opportunities to offer PrEP during healthcare visits to populations at risk, including BMSM [33]. Generally, socio-structural barriers (e.g., stigma, racism, homophobia, poverty) are major impediments to PrEP use among BMSM, thereby exacerbating HIV-related disparities in the South. These challenges therefore call for multilevel interventions to control the HIV epidemic and promote HIV prevention among BMSM in the South.

## 3. Impact of COVID-19 on Uptake and PrEP-related Research among BMSM

Although the EHE initiative was welcomed by scientists, funders, politicians, and other key stakeholders, the emergence of the coronavirus (COVID-19) pandemic created unforeseen challenges to meeting EHE goals. Given the nature of the virus, governments across the globe enacted mitigation measures, such as physical distancing and lockdowns, to curb the spread of the virus. Recent studies showed that COVID-19 negatively impacted HIV care and prevention services [2,34,35,36]. A national survey of MSM was conducted in April 2020; of 204 MSM who reported PrEP use, more than half (68.6%) reported not having any challenges getting a PrEP prescription, 8.8% had problems getting PrEP prescriptions, and 22.5% did not try to access PrEP at all [34]. Importantly, most participants in the survey were non-Hispanic White and had private health insurance, which may have facilitated their access to PrEP during the COVID-19 shutdown [34]. Similarly, Santos and colleagues reported that COVID-19 interrupted most MSM’s access to PrEP across the countries sampled (including U.S.) [35]. These findings showed the impact of COVID-19 on MSM access to PrEP and this may potentially increase health inequities among Black individuals in the South.

Furthermore, COVID-19 pandemic restrictions have also affected PrEP-related research in the Southern U.S. We conducted a rapid review of scientific literature (using PubMed and Google Scholar) of empirical studies on PrEP use among BMSM in the U.S. South published between January 2019 and May 2021 to specifically identify any studies published since the EHE initiative was announced as well as during COVID-19 pandemic. Of over 250 articles sampled, only 51 articles reported data partially or wholly collected in the South among BMSM. Although the articles were published between 2019 and 2021, most of the data reported were collected between 2012 and 2017, with only a few studies reporting data from 2018 and 2019 and no studies reporting data collected in 2020 or 2021. Although there is often a lag between data collection and dissemination, this shows the need for more recent data to adequately inform PrEP efforts among BMSM in the South. Since the declaration of EHE initiative in 2019 to eradicate the HIV epidemic within 10 years, few HIV studies have been conducted that focus specifically on BMSM [37]. While COVID-19 disruptions exacerbate the existing health disparities and barriers that inhibit access to PrEP, there is generally a dearth of data on PrEP uptake, retention, and adherence among BMSM in the U.S. [34,35,37,38].

In addition, some scholars have noted that the production of knowledge in the field of public health has mostly been rooted in biomedical research and theories, and it is yet to adopt intersectional and interdisciplinary approaches especially for HIV-related research among BMSM to address health disparities in U.S. [2,37,39,40,41]. The advancement of studies and interventions that adopt intersectional and interdisciplinary approaches to investigate health disparities, PrEP use, and adherence among BMSM is critical to ending the HIV epidemic in U.S. [34,38,42,43]. For example, HPTN 073 adopted intersectional approaches (e.g., addressing contextual and structural factors that impede on PrEP initiation and adherence among BMSM) to reach BMSM and showed high PrEP initiation among this group at risk of acquiring HIV [38,42]. It is therefore imperative for future studies to adapt intersectional approaches to have a better understanding of barriers and facilitators of PrEP use among BMSM in the South.

## 4. Conclusions and Key Recommendations

Historically, BMSM are disadvantaged within the health systems and underrepresented in HIV-related research. Intersectional stigma, racism, homophobia, and poverty must be addressed to improve uptake of PrEP among BMSM in the South. Additionally, there is an urgent need for PrEP promotion to reduce stigma surrounding this prevention strategy and address the myths and misinformation about PrEP, especially in BMSM community. Healthcare systems’ strengthening, such as healthcare providers’ training, awareness of systemic racism, and sensitization about sexual identity, must be prioritized. In agreement with some scholars [37,38], we recommend that any tailored programs or interventions that seek to address health disparities and contextual and structural factors inhibiting BMSM uptake of PrEP should adopt intersectional and interdisciplinary approaches for better understanding of the problems in order to develop culturally acceptable multilevel interventions. To achieve optimal improved health outcomes among BMSM, the community must be well represented in scientific research or programs on the issues that affect them. Furthermore, the rapid expansion of telehealth services during the COVID-19 period may offer increased opportunity to scale-up PrEP through telehealth interventions/services (e.g., Nurx©, Plushcare©), especially if in-person services remain limited due to pandemic precautions. Accelerating the expansion of PrEP for BMSM in the South (especially those living in rural areas) is critical to achieving the strategic goals of the EHE initiative. Additional studies are also needed to investigate the impact of COVID-19 on HIV-prevention services among BMSM to understand how this community has been affected by the current pandemic as well as to co-develop culturally acceptable multilevel interventions to reduce the identified challenges using intersectional and interdisciplinary approaches.

## Figures and Tables

**Table 1:** Estimated number of MSM with PrEP indications in the South by transmission-risk group, 2015.

States	Total Number of all Adults	Total Number of MSM	% of Total (MSM)
Alabama	11,840	7860	66.4%
Arkansas	4610	3350	72.7%
Delaware	4010	2390	59.6%
District of Columbia	13,820	8850	64.0%
Florida	115,200	73,570	63.9%
Georgia	35,700	25,330	71.0%
Kentucky	12,190	9100	74.7%
Louisiana	13,390	8380	62.6%
Maryland	27,390	15,700	57.3%
Mississippi	5010	3480	69.5%
North Carolina	29,820	21,160	71.0%
Oklahoma	9140	7170	78.4%
South Carolina	9040	6040	66.8%
Tennessee	22,880	15,530	67.9%
Texas	117,180	86,020	73.4%
Virginia	32,380	22,490	69.5%
West Virginia	3060	2220	72.5%
Total	466,660	318,640	68.3%

Source: extracted from the CDC data presented by Smith et al., 2018 [8].

**Table 2:** Estimated number of BMSM with PrEP indications in the South by race/ethnicity, 2015.

States	Total Number of Black/African Americans Adults	Total Number of BMSM	% of Total (BMSM)
Alabama	8440	5290	62.7%
Arkansas	2430	1640	67.5%
Delaware	2140	1070	50.0%
District of Columbia	10,010	5260	52.5%
Florida	48,860	23,800	48.7%
Georgia	26,750	18,280	68.3%
Kentucky	4620	3090	66.9%
Louisiana	9640	5640	58.5%
Maryland	20,740	11,110	53.6%
Mississippi	4000	2750	68.8%
North Carolina	19,080	12,740	66.8%
Oklahoma	2160	1610	74.5%
South Carolina	6210	3950	63.6%
Tennessee	13,450	8890	66.1%
Texas	43,270	27,490	63.5%
Virginia	19,990	12,700	63.5%
West Virginia	510	130	25.5%
Total	242,300	145,440	60.0%

Source: extracted from the CDC data presented by Smith et al., 2018 [8].

**Table 3:** Number of PrEP prescriptions in Southern States in 2017.

States	Number of People Who Could Potentially Benefit from PrEP	Number of People Who Were Prescribed PrEP in 2017	% of PrEP Prescriptions
Alabama	11,840	899	7.6%
Arkansas	4610	391	8.5%
Delaware	4010	N/A	N/A
District of Columbia	13,820	1869	13.5%
Florida	115,200	7594	6.6%
Georgia	35,700	2656	7.4%
Kentucky	12,190	N/A	N/A
Louisiana	13,390	1078	8.1%
Maryland	27,390	2015	7.4%
Mississippi	5010	363	7.3%
North Carolina	29,820	1798	6.0%
Oklahoma	9140	481	5.3%
South Carolina	9040	590	6.5%
Tennessee	22,880	N/A	N/A
Texas	117,180	6436	5.5%
Virginia	32,380	N/A	N/A
West Virginia	3060	N/A	N/A

N/A, data not reported. Source (The data in Table 3 were extracted from Rawlings and Parham-Hopson, 2021, and we calculated the percentage of each Southern state to show the trends in number of adults who were prescribed PrEP in 2017 compared to those who could benefit from it): extracted from the CDC data presented by Rawlings and Parham-Hopson, 2021 [3].

## Data Availability

Not applicable.
